# Bio-psycho-socio-demographic and Obstetric Predictors of Postpartum Depression in Pregnancy: A prospective Cohort Study

**Published:** 2014

**Authors:** Fatemeh Abdollahi, Samad Rohani, Ghazali Shariff Sazlina, Mehran Zarghami, Md Zain Azhar, Munn Sann Lye, Farideh Rezaiee Abhari, Zohreh Majidi, Soghra Mozafari

**Affiliations:** 1 Assistant Professor, Department of Public Health, School of Health, Mazandaran University of Medical Sciences, Sari, Iran.; 2 Associate Professor, Department of Family Medicine, School of Medicine and Health Sciences, University Putra Malaysia, Serdang, Malaysia.; 3 Professor, Psychiatry and Behavioral Sciences Research Center, Addiction Institute AND Department of Psychiatry, Mazandaran University of Medical Sciences, Sari, Iran.; 4 Professor, Department of Psychiatry, School of Medicine and Health Sciences, University Putra Malaysia, Serdang, Malaysia.; 5 Professor, Department of Community Health, School of Medicine and Health Sciences, University Putra Malaysia, Serdang, Malaysia.; 6 Lecturer, Department of Midwifery, School of Nursing and Midwifery, Mazandaran University of Medical Sciences, Sari, Iran.; 7 Health Care Provider, Health Center of Behshahr, Mazandaran University of Medical Sciences, Sari, Iran.

**Keywords:** Cohort Study, Prediction, Postpartum Depression, Risk Factors

## Abstract

**Objective:** There are various attempts to confirm variables that could predict postpartum depression in advance. This study determined antenatal risk factors for postpartum depression in women at risk of developing this disorder.

**Methods:** A prospective cohort study was conducted with 2279 eligible women who attended at Mazandaran province’ primary health centers from 32-42 weeks of pregnancy to eighth postpartum weeks. The women were screened for symptoms of depression using the Iranian version of Edinburgh Postnatal Depression Scale. An Edinburgh Postnatal Depression Scale score of > 12 indicated possible postpartum depression. Univariate and multiple logistic regression models were used for data analysis.

**Results: **A total of 2083women during 32-42 weeks of gestation participated in this study and were followed up to 8-week postpartum. Four hundred and three (19.4%) mothers yielded scores above the threshold of 12. Depression and general health state in pregnancy based on Edinburgh Postnatal Depression Scale (OR = 1.35, CI = 1.3-1.4) and General Health Questionnaire-28 (OR = 1.03, CI = 1.01-1.04), respectively were significant independent antenatal risk factors of depression symptoms at 8-week postpartum. Mothers who lived in nuclear families (OR = 1.38, CI = 1.04-1.84), whose husbands had lower educational status (OR = 0.95, CI = 0.91-0.99), and with delayed prenatal care (OR = 1.01, CI = 1.001-1.03) were more susceptible to postpartum depression.

**Conclusion:** A comprehensive antenatal assessment focused on psychiatric problems, environmental and obstetric factors would benefit pregnant women in the prevention of postpartum depression.

## Introduction

During the past two decades, postpartum depression (PPD) has been considered as a significant health issue for mothers and family members (1). The risk of depression during 3-6 months following birth as compared to other times of life is increased by three-fold (2). Depression affects 10-15% of postpartum women in Western countries (3). Cross-sectional studies have shown the occurrence of an increasingly high rate of PPD within diverse places of Iran (17.5 to 35.0 percent) (4-6). Cognitive, emotional and social developments as well as behavioral difficulties are more common in children of mothers with PPD even with subclinical conditions (7-9).

There have been several researches, meta-analyses and systematic reviews attempting to build and validate a predictive index capable of determining the risk of development of PPD in advance (10-12). However, the results were not conclusive and no single hypothesis is able to elucidate this phenomenon. Still it is uncertain whether socio-demographic, psycho-sociological, biological, or obstetric factors make women vulnerable to postpartum depression (13). Some of these risk factors are amendable to intervention. Thus, screening women who are at risk during pregnancy would help to decrease the probability of getting PPD prior to its development. In order to determine the association between PPD and the variety of risk factors that could predispose women to depression, we hypothesized that complex interactions between demographical, psychological, sociological, biological, and prenatal factors such as obstetrics and gynecologic complications are implicated in PPD in an Iranian population.

## Materials and Methods

**Figure 1:**
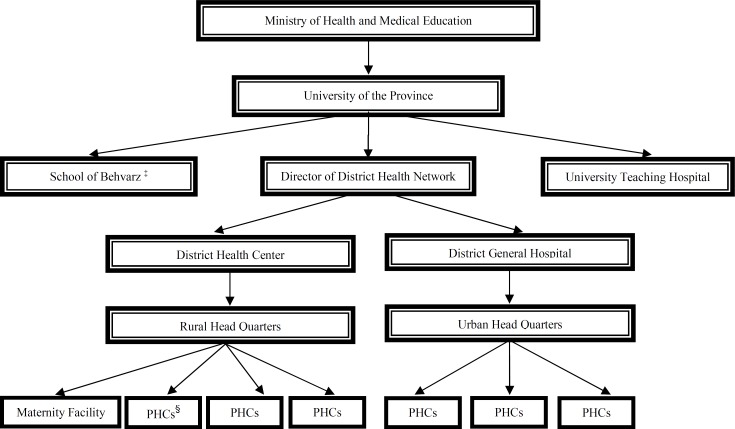
Organization chart of health care delivery system of the Islamic Republic of Iran, (Mazandaran Annals Statistics, 2007; Mesgari et al., 2008

^†^ Diploma level health workers, working in rural PHCs; ^‡^ Diploma level health workers, working in urban PHCs; § Primary health center

This study was conducted using the data of an ongoing longitudinal study of 16-45 year-old women who were attending primary health centers (PHCs) in Mazandaran University of Medical Sciences (MAZUMS) in Sari, Iran from January to July2009. G-power software for logistic regression was used to estimate the sample size (14). In each province in Iran, there are PHCs with well-designed health programs in each city (Figure 1). Approximately 98% of pregnant women receive services from the antenatal health centers in the PHCs of Mazandaran province (15). All programs provided by PHCs are free and mostly for the low and middle-income families. 

The researchers and educated health care practitioners distributed the self-report questionnaires among eligible women who attended the PHCs. The type of sampling that was used in this study was convenience sampling method. Literate women aged 16-45 years old who were at their 32-42^nd ^weeks of gestation were eligible for entering this study. Women receiving pharmacological treatments for psychiatric problems were excluded. The questionnaires of potential risk factors in the third trimester of pregnancy were used to estimate the risk of PPD at 8 weeks postpartum. A telephone call was made 2-3 days prior to each meeting as a reminder. The study was approved by the MAZUMS and University Putra Malaysia (UPM) and all participants signed an informed consent form containing a specific code.

For collecting the data in this study, the following standard instruments were used.

*Edinburgh Postnatal Depression Scale* *(EPDS):* It is a 10-item Likert self-report questionnaire that takes 10 minutes or less for mothers to complete it. A mother selects one of four possible responses (“no, not at all” to “yes, quite often”) according to her feelings within the past seven days (16). The scores of 12 or more was considered as cut-off point in this study as most studies indicate that mothers with scores of 12 or greater were prone to develop depression and have been referred to psychiatric visits. The EPDS has been validated for antenatal and postnatal women (16-18).

*Premenstrual Syndrome Questionnaire (PMSQ): *This self-report questionnaire consists of 22 items of physical and psychological symptoms within 7 to 10 days prior to menstruation. The responses are ranked on a scale from 0 (lack of symptoms) to 3 (severe symptoms), and total scores show the severity of syndrome (19). In order to have a positive symptom, patient must show at least one somatic and psychological sign in the second part of menstruation cycle lasting for four days and for three consecutive cycles, and to be severe thus, is necessary to seek the physician for treatment (19, 20).

*Social Support Appraisals Scale* *(SSA): *It is a 23-item self-report instrument created by Vaux et al. (1986) (21). Each Likert item has four responses from strongly agree (scored as one) to strongly disagree (scored as four). Then all items are summed up to obtain a total score. In this evaluation, lower scores demonstrate stronger social support. The SSA is a good predictor of social support and also psychological well-being such as depression and loneliness (22). 

*Network Orientation Scale (NOS)* *(* 23 *)* *:* Vaux (1985) designed a 20-item self-report instrument called Network Orientation Scale to measure negative network orientation and to assess the individual’s unwillingness to keep, look after, or employ the kind of support that she has (24). Each Likert item has four responses from strongly agree (scored as one) to strongly disagree (scored as four). All items are calculated to obtain a total score. The highest score demonstrates more negative orientation.

*General Health Questionnaire* *(GHQ-28):* The GHQ is a 28-item self-report questionnaire designed by Goldberg (1972) to assess the presence of psychiatric distress related to general medical illness (25) . Each item is rated from zero to three, with higher scores indicating a higher probability of depression and socio-psychosomatic symptoms (26) . The score of higher than 21 is used for predicting psychiatric disorders within Iranian population (27) .

*ENRICH Marital Inventory* *:* This self-report questionnaire consists of 14 subscales; items 6 to15 is concerned with marital satisfaction. It is a Likert tool that respondents choose from strongly agree to strongly disagree (28). The ENRICH is scored by summing up the scores of all items ranging from zero to four. The higher score indicates better the marital relationship (29). In Mahdavian’s study, correlation coefficient of marital satisfaction subscale of ENRICH was 0.85 (30).

*Life Events Rating Scale* *(LEQ):* This self-report questionnaire is designed by Holmes and Rahe (31). A study by Brahani et al. on employees of Tehran, Iran hospital modified this scale to 42 items (32). The response is based on two forms; yes-no, and another one is ranked on a Likert scale from 0 (not agree) to 3 (strongly agree) based on intensity of event (33). In this study, the researcher utilized the first form of scoring (yes-no). The total number of individual events indicates the individual score.

PMSQ, SSA, GHQ, ENRICH, LERS and EPDS have been used before in Iranian population, and their validity has been established (5, 20, 27, 29, 32, 34, 35). Content validity of Persian version NOS, was tested and used after correction. 

The participants provided information on demographics, socio-economic, mental health, hormone related conditions, all kinds of abuse (physical, psychological and sexual) before and during pregnancy, and obstetric and gynecological data using a standard questionnaire that was designed by investigators after reviewing literature on risk factors of PPD. Ten specialists on psychiatric, public health, obstetrics, biostatistics and epidemiology in MAZUMS and UPM validated the content of the questionnaires.

The socio-demographic and economic information included mother’s age, age at marriage, parity, gravida, level of highest education (lower secondary; ≤ 9, upper secondary; 10-12 and completed high school > 12), employment status (housewife, employed and student), husband’s level of highest education and employment status (business, government servants, farmer, student and others), family structure (extended and nuclear), housing condition (renting and own house) and monthly income (low < 3,500,000 IRR (Iranian Rials Rate), medium = 3,500,000-4,500,000 IRR and high > 4,500,000 IRR). Information on family history of depression and psychosis, history of psychosis, previous postpartum depression, history of depression in the first and second trimester of pregnancy, irritable mood before menstruation, mood instability due to oral contraceptive pills (OCP) and mood instability at puberty, medical problems, infertility, any complications during previous and this pregnancy and after birth, gestational age at the first antenatal care visit, gestational age at the time of delivery, number of antenatal care visits, unwanted pregnancy, participation in health education programs, weight gain, mode of delivery, place of delivery, breastfeeding status, birth weight and gender of infant, and neonatal morbidity. In this study, there were 24 questions that capture information on whether subjects were being abused or witnessed abuse. Yes (scored 1), No (scored 0) answers were added up to create a total score ranging from 0 to 24, with greater scores demonstrating more risk. Most information (demoghraphic data, life events, social support and so on) were collected in the base of the study during the 32-42 weeks of pregnancy, and other data including some obstetric factors were collected at eight weeks after birth.

The questionnaire was tested with a sample of 60 healthy unselected pregnant mothers who attended the PHCs and reliability of all questionnaires was estimated with Cronbach’s alpha ranged between 0.42 and 0.92. 

Sequential logistic regression analyses were performed using SPSS for Windows 20.0 (SPSS Inc., Chicago, IL, USA) to predict depressive symptomatology at 8-week postpartum as measured by EPDS scores > 12 and develop an antenatal predictive tool to forecast the PPD. In the univariate analysis, variables were tested in the model one at a time and the corresponding odds ratio (OR) and 95% confidence intervals (CI) were derived. In the hierarchical multiple logistic regression, variables were entered into the model in the following sequential order: mental health, psychosocial, obstetric and gynecological, socio-demographic, and hormone-related factors significant variables were tested and retained in the model if the P-value for the β-estimate was 0.05 or less as derived from the Wald statistic.

## Results

During the study 9,187 women with 32-42 weeks of gestation registered at 308 PHCs in the province. Among 214 PHCs who took part in the study, 2,626 women were eligible and approached in the study. Of those, 2,359 (89.8%) volunteered and consented and of these 2,279 (96.6%) completed the questionnaires during 32-42 weeks of pregnancy. We used convenient method for sampling. The total numbers of women who participated at both pregnancy and eight weeks postpartum were 2,083 (91.4%). 

The sample was representative of Mazandaran childbearing population as far as age and parity are concerned. A comparison was made between pregnant mothers who attended all 308 health centers in 2009 for prenatal care (42,000 mothers) and those 2083 mothers in our sample. The average age was 25.9 years vs. 26.07 years, respectively; and parity was 1.6 vs. 1.4, respectively (p > 0.05). 

Two thousand and two hundred seventy nine women from 32-42 weeks of pregnancy were followed up to ascertain the presence of depression and related risk factors at 8-week postpartum with response rates of 91.4% (2083). Based on EPDS, 21.4% (95% CI = 17.99-21.45) (445) of women had depression in late of pregnancy with a mean of 8.62 ± 4.9. A point prevalence of PPD at 8-week postpartum was 19.4% (95% CI = 20.04-23.63) (402) with a mean of 8.39 ± 4.9.

***Characteristics of the sample***

The mean age and marital age of 2083 women who completed the study were 26.07 ± 5.21 and 20.54 ± 4.1 years. The mean years of women's and their husbands' education were 10.65 ± 3.05 and 10.47 ± 3.2 years, respectively. The mean household income was 31732.53 ± 150195.15 IRR (approximately 317.32 USD) per month. The greater proportion of women were homemakers (96.3%) and living with husband who most of them had own business (68.1%), owned their own home (60.3%), and did not share a home with their extended family (72%).

The majority of women (60.4%) were primiparas. The ratio of male to female babies was 1:1. This study was conducted in rural and urban health centers with the proportion of 48.6% and 51.4%, respectively. 

Rituals mothers and their close relatives practiced after birth contain general, maternal, nutritional and neonatal practices. The number of cultural practice was ranged from zero to 27; with a mean of 14.11 ± 4 and maternal behaviors was the most common cultural practice in this study with a mean of 5.55 ± 1.89. Table 1 shows the obstetrics and hormonal characteristics of mothers under study. 

***Risk factors for postpartum depression***

In univariate analysis, most variable showed statistically significant effects in the line of previous studies results. Variables considered for the model comparing women with depression to women without depression (based on EPDS) included age at marriage, family structure, husband’s education, first antenatal visit, medical diseases, anemia, obstetric complications in previous pregnancies, recurrent urinary infection, un-planned pregnancy, no health education, depression in late of pregnancy, depression and anxiety in 1^st^ and 2^nd^ trimesters of pregnancy, previous PPD, family history of depression, low general health status, no social support during pregnancy , social isolation during pregnancy, increased number of life events, marital satisfaction during pregnancy , and increased rate of abuse that were entered into multiple hierarchical regression to explore the independent variables that predispose women to PPD (Table 2) (Table 3).

In the final model, high score of EPDS in pregnancy and psychiatric distress based on GHQ were significant mental health indicators for prediction of depressive symptoms through 8-week by odds ratio of 1.35 (95% CI = 1.3-1.4) for high EPDS score and 1.03 (95% CI = 1.01-1.04 for psychiatric disorder based on GHQ. Also, living in extended families and delay in antenatal care increased the odds ratio of an elevated postpartum EPDS scores. Similarly, increased level of husband’s education had a statistical significantly risk reduction effect. Adjusted odds ratios and 95% confidence intervals for the independent predictors retained in the final model are presented in table 4.

**Table 1:** Obstetrics and hormonal characteristics of women attending Mazandaran province primary health centers who followed up 8-week postpartum, 2009 (n = 2083)

**Variables**	**n (%)**	**Variables**	**n (%)**
**Number of prenatal visits**		**Type of delivery**	
**Less than 9**	1238 (59.5)	Emergency cesarean section	578 (27.3)
**9-12**	519 (25)	Elective cesarean section	389 (18.7)
**More than 12**	323 (15.5)	Vaginal and instrumental delivery	1113 (53.5)
**Gestational age at time of delivery (weeks)**		**Breast feeding status at three ** **months**	
**Premature (< 37)**		Exclusive breast feeding	1846 (90.6)
**Term (≥ 37)**	355 (17.1)	Bottle feeding	37 (1.8)
	1723 (82.9)	Mix feeding	154 (7.6)
**Sex of infant**		**Irritable mood at puberty**	
**Male**	1045 (50.2)	Yes	233 (11.2)
**Female**	1035 (49.8)	No	1850 (88.8)
**Birth weight(grams)**		**Irritable mood before menstruation**	
**LBW (< 2500)**	84 (4)	Yes	364 (17.5)
**No-LBW (≥ 2500)**	1994 (96)	No	1719 (82.5)
**Neonatal morbidity**		**Mood instability due to OCP** †	
**Yes**	251 (12.1)	Yes	579 (83.5)
**No**	1829 (87.9)	No	114 (16.5)

† Oral contraception pill

**Table 2:** Socio-demographic and obstetrics factors associated with depression at 8-week postpartum using simple logistic regression (n = 2083)

**Risk factors **	**EPDS ≤ 12**	**EPDS > 12**	**OR**	**95% (CI)**	**P-value**
**Age at marriage (years)**					
**≤ 24**	1449	230	0.69	0.52-0.92	0.010
**≥ 25**	0305	172			
**Family structure**					
**Extend**	1228	272	1.32	1.04-1.67	0.020
**Nuclear**	0451	132			
**Husband education (years)**	1679	404	0.96	0.93-0.99	0.020
**First antenatal visit (weeks)**	1679	404	1.02	1.01-1.04	0.001
**Obstetrics complications in previous pregnancies**					
**Yes**	0361	073	1.64	1.15-2.34	0.005
**No**	0252	084			
**Anemia**					
**Yes**	0102	056	1.61	1.16-2.24	0.004
**No**	1527	348			
**Medical diseases**					
**Yes**	0197	063	1.39	1.02-1.89	0.030
**No**	1482	341			
**Recurrent urinary infection **					
**Yes**	0184	339	1.55	1.14-2.11	0.005
**No**	1495	065			
**Planned pregnancy**					
**Non-planned**	0320	108	1.55	1.20-1.99	0.001
**Planned**	1353	294			
**Health education**					
**No**	0247	077	1.36	1.02-1.80	0.030
**Yes**	1426	326			
**Puerperal complications **					
**Yes**	0456	252	1.58	1.26-1.99	0.001
**No**	1214	150			

**Table 3:** Psycho-social factors associated with depression at 8-week postpartum using simple logistic regression  (n = 2083)

**Risk factors **	**EPDS ≤ 12**	**EPDS > 12**	**OR**	**95% (CI)**	**P-value**
**Depression in 32-42 weeks of pregnancy based on EPDS** †					
**Yes**	0171	272	1.39	1.34-1.43	0.001
**No**	1507	131			
**Depression in 1** ^st^ **, and 2** ^nd^ ** trimesters of pregnancy**					
**Yes**	0175	098	2.75	2.08-3.62	0.001
**No **	1504	306			
**Anxiety in 1** ^st^ **, and 2** ^nd^ ** trimesters of pregnancy**					
**Yes**	0952	155	2.10	1.68-2.62	0.001
**No **	0727	249			
**Previous PPD** ‡					
**Yes**	0041	024	2.70	1.57-4.62	0.001
**No**	0623	135			
**Family history of depression**					
**Yes**	1571	343	2.58	1.85-3.61	0.001
**No **	0108	061			
**General health status from GHQ** §					
**Not-Healthy (> 21)**	1023	128	3.36	2.66-4.23	0.001
**Healthy (≤ 21) **	0656	276			
**Social support during pregnancy from SSA** ||					
**Low (≥ 51)**	0469	084	2.009	1.48-2.71	0.001
**Medium (45-50)**	0820	179	1.21	0.91-1.61	0.17
**High (≤ 44) **	0389	140			
**Social isolation during pregnancy from NOS** ¶					
**Low (≥ 51)**	0462	084	1.98	1.47-2.67	0.001
**Medium (45-50)**	0791	166	1.15	0.86-1.53	0.32
**High (≤ 44)**	0423	153			
**Number of life events from LERS** ††					
**≥ 4**	0633	103	2.08	1.59-2.71	0.001
**2-3**	0497	115	1.42		0.01
**≤ 1**	0549	186		1.06-1.9	
**Marital satisfaction during pregnancy from MI** ‡‡					
**Low (≤ 23)**	0742	130	1.60	1.22-2.1	0.01
**Medium**	0530	123	0.75	0.57-0.98	0.4
**High**	0406	151			
**Postnatal parenting self-efficacy from PES** §§					
**Low (≤ 7)**	0447	090	1.62	1.19-2.19	0.002
**Medium (7.01-8.91)**	0775	170	1.08	0.82-1.44	0.5
**High (≥ 9)**	0377	123			
**Abused**	1679	404	1.11	1.06-1.16	0.001

† Edinburg Postpartum depression scale;

‡ Postpartum depression;

§ General Health Questionnaire;

|| Social Support Appraisal

¶ Network Orientation Scale;

†† Life Event rating Scale;

‡‡ Marital Satisfaction Index;

§§ Parental Expectation Survey

**Table 4:** Demographic, psychosocial, obstetric and mental health factors and risk of PPD at 8-week postpartum using hierarchical multiple logistic regression (n = 1449)

**Risk factors**	**Β**	**SE**	**Adjusted OR**	**95% CI**	**P-Value**
**Depression in 32-42 weeks of pregnancy based on EPDS** †	-0.30	0.010	1.35	1.30-1.40	0.001
**General health status from GHQ** ‡	-0.02	0.007	1.03	1.01-1.04	0.001
**Husband education**	-0.04	0.030	0.95	0.91-0.99	0.030
**Family structure**	-0.32	0.140	1.38	1.04-1.84	0.020
**Gestational age at first antenatal visit**	-0.01	0.009	1.01	1.001-1.03	0.030
**Constant**	-5.18				

† Edinburg Postpartum depression scale;

‡ General Health questionnaire

## Discussion

Our findings challenge the idea that claims PPD to be a principally developed Western nations’ problem. In the present study, the PPD prevalence of 19.4% (95% CI = 20.04-23.63) at 8-week postpartum was comparable with cross-sectional studies of Iranian populations (17.5%-36%)   (4-6) . Our findings underscore the risk of PPD in developing countries (21-36%) (36, 37)  and found the prevalence to be high in comparison to other reports of populations in the developed countries that 10-15% of women were affected by PPD   (38-40) . However, comparisons are compromised by the variations in the timing of follow-up and using different EPDS thresholds.

Our findings can facilitate early detection of women at risk for PPD based on causal risk factors including psychological and environmental, combined with obstetric and demographic factors during pregnancy. Multivariate model illustrated that presence of depression in 32-42 weeks of pregnancy based on EPDS, high psychiatric distress status from GHQ-28, husband with lower educational status, living in extended family and delay in antenatal visit were independently effect on developing PPD at two months post-delivery in the Iranian population.

Psychiatric distress and history of depression in pregnancy have been consistently found to be an important predictor of PPD (12, 41-43). Meta-analyses and other studies regarding risk factors for PPD found that depression and anxiety during pregnancy are strongest risk factors for increasing the chance of PPD (10). Further substantiating this finding, in a longitudinal follow-up study (n = 1618), Verkerk et al. investigated antenatal predictors of the occurrence of PPD in high risk (EPDS > 11) and low risk (EPDS < 8) groups from mid-pregnancy to 12 months of postpartum. Depression symptomatology during pregnancy was found to be the strongest risk factors that predisposed mothers to the development of PPD in the two groups (44). Thus, these findings demonstrated the importance of antenatal depression assessment and close monitoring of women who gained high scores of EPDS during pregnancy at postpartum period (43).

In the multivariate model, there was no evidence of association between depression in the 1^st ^and 2^nd ^trimester of pregnancy and PPD. Although in the univariate analysis this association was statistically significant. One possibility may be considered for this negative finding; when two variables are entered into the model to measure the same concept, the variable that have more important role to predict PPD is the one that is retained as significant (45).

The data of the current study also showed mothers whose husbands with higher level of education were less likely to go through PPD. As for husbands’ demographic characteristics, a Pakistani study by Rahman and Creed yielded a significant positive association between uneducated husbands and persistent PPD for one year after birth (46). Furthermore, a review study in Asian countries revealed that having an unemployed or uneducated husband is a risk factor for PPD (47). The association between husband’s education and PPD could be related to the fact that husbands with higher level of education provided more support during a specific period such as postpartum. The finding of the current study was in agreement with that of Rahman et al. who demonstrated that when compared with living in nuclear families, mothers living in extended families were associated with a slightly increased risk of PPD (48). In extended families such as families in the United Arab Emirates, the sons’ mother in-law traditionally has influence over new mothers and the weak relationship with husbands’ family, especially mother in-laws may result in marital conflict and cause high risk of PPD (4, 41, 49). Nowadays, unsupportive husbands often distress mothers who have to adapt to familial relationships in various value structures. Chinese mothers with a history of PPD were found to have encountered conflicts in trying to adapt to a new way of life by being accommodating and amenable, which is essential for the stability and harmony of family while longing to assert their modern values such as independence and individuality (50). 

Concerning obstetrics data, delay in prenatal visit was the only factor that increased the risk of PPD in the final model. In a well-designed case-control study conducted by Josefsson et al. higher number of prenatal visits was the most significant risk factor PPD (51). These findings revealed that obstetrics related problems may render predisposes women to PPD. However, the existing evidences are incompatible and the studies' effect size was small (10).

In contrast to the current literatures, our study did not show that psychosocial difficulties during pregnancy significantly predict PPD (37, 52). Although, it is not easy to give an explanation for this, it may be that almost everyone has some support from others in the Iranian cultures (4).

The rate of 19.4% for depression vulnerability among women at 8-week postpartum women indicates a high prevalence of PPD among Iranian population. Prevalence measurements are important for identifying health problems and disease burden as well as being useful in planning health service delivery, human and other resources, as well health care program assessment (53, 54).

A wide range of potential PPD risk factors were assessed in this prospective study. Multivariate analysis revealed that the risk of PPD increases in the cases of psychological and environmental factors combined with demographical factors. Thus, this particular group of depressed mothers should be considered a high risk group that needs special attention. 

Few studies have investigated the antenatal risk factors for PPD in the Iranian populations. Prospective design is an important strength of the present study. This population-based research has analyzed various PPD risk factors in a large sample size using validated screening instruments.

Although EPDS is a screening tool, it would have been ideal to confirm mothers with increased EPDS scores with the use of Diagnostic and Statistical Manual of Mental Disorders, 4^th^ edition (DSM-IV) or Diagnostic Interview Schedule-III-Revised (DIS) (55). Selection bias may occur as the women participated voluntarily in the study. Besides, the present study was limited by excluding illiterate mothers from the study and it was not possible to compare characteristics of participants with those who did not participate or were not eligible to enter this study.

Determination of PPD risk factors are important in understanding the mechanisms in which a mother may become depressed, and can assist in developing interventions resulting in efficient and productive treatments. The findings of this study suggest an essential opportunity for healthcare providers to predict PPD. They should alert women to the potential risk factors encourage them to report early symptoms of PPD. Early screening and intervention can prevent the serious effects of PPD and should be incorporated into clinical assessment protocols. 

## Footenote

**Declaration of interest:** None.
